# Comparative Evaluation of Sonic and Ultrasonic Activation of 3 Post Space Irrigants on Push-Out Bond Strength of Fiber Posts Luted With Dual-Cure Resin Cement: Protocol for an In Vitro Study

**DOI:** 10.2196/76907

**Published:** 2026-01-28

**Authors:** Apurva Wamane, Aditya Patel

**Affiliations:** 1 Department Of Conservative Dentistry And Endodontics Sharad Pawar Dental College And Hospital Wardha India; 2 Department of Conservative Dentistry and Endodontics Datta Meghe Institute of Higher Education and Research Sharad Pawar Dental College and Hospital Wardha India

**Keywords:** fiber post adhesion, bond strength, post space irrigants, ultrasonic vs sonic activation, 5% calcium hypochlorite, 17% ethylenediaminetetraacetic acid, 17% EDTA, dual-cure resin, smear layer removal

## Abstract

**Background:**

For long-term longevity and proper function, endodontically treated teeth must be restored appropriately. Fiber posts luted with dual-cure resin cement are frequently used because of their improved biomechanical and adhesive properties. However, achieving a strong binding between the fiber post and root canal dentin is difficult due to the physical constraints of the root canal and the presence of a smear layer. Bond strength may be increased by post space irrigation with solutions, such as 5% calcium hypochlorite, 17% ethylenediaminetetraacetic acid (EDTA), and saline, that have been activated using sonic or ultrasonic techniques. This study compares the effects of several activation techniques on the push-out bond strength of fiber posts luted with dual-cure resin cement.

**Objective:**

This study aims to evaluate how the push-out bond strength of dual-cure resin–cemented fiber posts in excised human mandibular single-rooted premolars is affected by the sonic and ultrasonic activation of 3 post space irrigants: 5% calcium hypochlorite, 17% EDTA, and saline.

**Methods:**

A total of 120 single-rooted mandibular premolars will be used in this in vitro investigation. A standardized procedure will be used to decoronate, instrument, and obturate the teeth. One of the 3 solutions, activated either sonically or ultrasonically, will be used to prepare and irrigate post spaces. Dual-cure resin cement will then be used to lute the fiber posts. Using a universal testing machine, push-out bond strength will be measured. The Tukey post hoc test and ANOVA will be used for statistical analysis of the data.

**Results:**

Ethics approval for this study was obtained from the institutional ethics committee of Datta Meghe Institute of Higher Education and Research. The study is self-funded. Sample collection of extracted human mandibular premolars began in June 2025, and 68 specimens have been collected as of submission. Specimen preparation is ongoing, with data collection projected to be completed by October 2025. Data analysis is scheduled for November 2025, and results are expected to be published in early 2026. It is anticipated that irrigant type and activation method will influence push-out bond strength, with 17% EDTA and 5% calcium hypochlorite, particularly under ultrasonic activation, potentially yielding higher values than saline.

**Conclusions:**

On the basis of previous literature, we expect that EDTA and calcium hypochlorite with ultrasonic activation may exhibit higher push-out bond strength.

**International Registered Report Identifier (IRRID):**

DERR1-10.2196/76907

## Introduction

### Background

Restoring endodontically treated teeth requires consideration of several factors, such as the position of the tooth in the dental arch, the type of restoration required, the amount of healthy tooth structure remaining, and the bond between the restoration and the natural tooth structure. When a significant amount of healthy tooth structure remains, the optimal approach often involves the use of a post and core followed by a final restoration [1,2].

In recent years, adhesive nonmetallic post systems, including glass fiber posts, fiber-reinforced posts, and polyethylene posts, have gained popularity because of their ability to preserve the remaining tooth structure during radicular preparation [3]. Glass fiber posts, which are clinically beneficial, offer a straightforward preparation process. When combined with adhesive cementation, this method produces a stronger bond, ultimately improving the clinical prognosis by enhancing biomechanical behavior under stress and reducing the likelihood of root fractures [4].

Following post space preparation, a smear layer containing abrasive debris, sealer residue, and gutta-percha can form on the dentin walls, which can prevent adequate bonding of the post to the tooth. Therefore, post space irrigation is essential to remove this debris and to provide benefits such as low toxicity, tissue dissolution, and antibacterial activity [5,6].

Achieving reliable bonding to root canal dentin can be challenging because of irregular canal geometry and differences in the physical and chemical properties of the luting materials. Additionally, any previous endodontic treatments may interfere with bonding during post cementation [7]. A strong bond between the fiber post and the root canal walls is critical because debonding is often the primary cause of fiber post failure in clinical settings [8]. The development of self-adhesive cements has simplified the adhesion process, improving bonding to radicular dentin and making it less technique sensitive [9].

Treatment irrigating solutions can alter the chemical and structural composition of dentin, potentially affecting its permeability and solubility [10]. These changes may enhance or hinder the ability of adhesive agents to bond effectively to the dentin [11]. Specifically, calcium hypochlorite has been shown to increase calcium content while decreasing carbon content, which may promote mineralization and lead to the formation of calcium phosphate and calcium carbonate crystals on the dentin surface, thereby strengthening the bond between the adhesive and dentin [12].

Ethylenediaminetetraacetic acid (EDTA), a bivalent cationic substance that chelates calcium ions, is frequently used in endodontics to remove the smear layer. This process removes noncollagenous proteins and hydroxyapatite, thereby facilitating better penetration of resinous agents and resulting in a thinner hybrid layer without denaturation of collagen [13].

The activation of irrigants plays a crucial role in enhancing penetration and effectiveness. Sonic irrigation, operating at frequencies ranging from 1 to 6 kHz (SuperEndo Activator; Eighteeth Healthcare), generates less shear stress and, when paired with soft polymer tips, can prevent unnecessary dentin loss [13].

Ultrasonic agitation (Woodpecker UDS-K ultrasonic unit; Guilin Woodpecker Medical Instrument), operating at approximately 25,000 vibrations per second, enhances irrigant penetration into dentinal tubules through cavitation and acoustic streaming. Martin and Cunningham [14] highlighted its superior biological and chemical effects on root canal dentin surfaces.

The push-out bond strength test was chosen for its accuracy and reliability over shear and microtensile tests, especially when evaluating different root regions [12]. Adhesion of fiber posts to root canal dentin is complex because of anatomical and material-related challenges [4], with most failures occurring at the cement-dentin interface [15]. Previous studies have shown that 17% EDTA [16] and 5% calcium hypochlorite [17] significantly improve the bond strength of dual-cure resin–luted fiber posts.

In recent years, calcium hypochlorite ([Ca(OCl)_2_]) has emerged as a promising alternative to sodium hypochlorite for post space irrigation because of its higher release of available chlorine, improved antimicrobial action, and formation of calcium-rich precipitates on dentin surfaces. These precipitates enhance mineral deposition and may improve the chemical interaction between dentin and resin cement.

Ferreira et al [12] reported that calcium hypochlorite modifies the dentin surface by increasing calcium content and reducing carbon content, which can favor adhesive bond formation. In addition, Sheikh Ghahderijani et al [17] demonstrated that 5% calcium hypochlorite significantly improved push-out bond strength when used as a post space irrigant, making it a viable option for enhancing adhesion between fiber posts and root dentin.

Although several studies have evaluated the effect of different irrigants and activation methods on smear layer removal and post retention, the available evidence remains inconsistent. Some investigations have reported superior cleaning efficacy and bond strength with ultrasonic activation, while others found no statistically significant difference compared with sonic activation. These discrepancies may be attributed to variations in study design, irrigant concentration, activation duration, and tooth type. Moreover, many previous studies have focused on single irrigants or have not used standardized comparative designs, making it difficult to draw definitive clinical conclusions.

This study uses the push-out bond strength test, which has advantages over other laboratory methods, such as shear bond strength and microtensile testing. The push-out test applies a uniform stress along the post-dentin interface, more closely simulating the dislodgement forces experienced clinically. It also allows evaluation across different root regions, providing more clinically relevant data on the performance of irrigant-activation combinations. By addressing these methodological gaps, this study aims to provide a direct comparison of sonic and ultrasonic activation across multiple irrigants under standardized conditions.

However, the effects of sonic and ultrasonic activation of these irrigants have not been compared extensively in research. Therefore, to help increase the durability and efficacy of endodontic treatments, this study aims to assess and contrast the effects of sonic and ultrasonic activation of 5% calcium hypochlorite, 17% EDTA, and saline on the push-out bond strength of fiber posts luted with dual-cure resin cement.

### Research Question

This study was designed to evaluate the influence of different irrigant activation techniques on the adhesion of fiber posts to root canal dentin. The research question was as follows: Will the use of sonic and ultrasonic activation of 3 different post space irrigating solutions, namely, 5% calcium hypochlorite, 17% EDTA, and saline, affect the push-out bond strength of dual-cure resin–luted fiber posts in extracted human mandibular single-rooted premolars?

### Aim

The aim of this study is to compare the mean push-out bond strength (in megapascals [MPa]) of fiber posts luted with dual-cure resin cement after post space irrigation with 5% calcium hypochlorite, 17% EDTA, or saline when activated using either sonic or ultrasonic techniques in extracted human mandibular single-rooted premolars.

### Objectives

The objectives of this study are as follows:

First, to measure and compare the push-out bond strength (in MPa) of fiber posts following post space irrigation with 5% calcium hypochlorite, 17% EDTA, or saline, using sonic activation.

Second, to measure and compare the push-out bond strength (in MPa) of fiber posts following post space irrigation with 5% calcium hypochlorite, 17% EDTA, or saline, using ultrasonic activation.

Third, to determine what type of irrigant, activation method, or combination produces the highest mean push-out bond strength (in MPa).

### Null Hypothesis

The null hypothesis is that there is no statistically significant difference in mean push-out bond strength (in MPa) among any of the irrigant-activation combinations tested.

### Alternative Hypothesis

The alternative hypothesis is that at least 1 irrigant-activation combination produces a statistically significant difference in mean push-out bond strength (in MPa) compared with the others.

## Methods

### Eligibility Criteria

The inclusion and exclusion criteria are presented in Textbox 1.

**Inclusion and exclusion criteria of the research participants.**

**Inclusion criteria**

- Recently extracted permanent mandibular lower premolar teeth with a closed apex
- Teeth free of cavities, fractures, and previous endodontic treatment

**Exclusion criteria**

- Teeth with unusual shapes or visible cracks
- Teeth with abnormalities in their development
- Teeth with external and internal resorption

### Sample Size Estimation

The sample size was estimated with the following formula:

n1={(σ1^2^+σ_2_^2^/κ) (Z_1−α/2_+Z_1−β_)^2^} ÷ Δ2

Where n_1_=sample size of group 1; n_2_=sample size of group 2; σ_1_=SD of group 1; σ_2_=SD of group 2; Δ=difference in group means; κ=ratio of n_2_ to n_1_ (n_2_/n_1_); Z_1−α/2_=2-sided *Z* value (eg, *Z*=1.96 for 95% CI); and Z_1−β_=0.84 (80% power).

σ=pooled SD, calculated as follows:

σ = Ö(σ_1_^2^ + σ_2_)/2=Ö[(10.19)^2^ + (10.04)^2^]/2 = 10.12

Where Δ=expected mean difference in push-out bond strength (49.08 − 39.82 = 9.2649.08 – 39.82 = 9.2649.08 − 39.82 = 9.26 MPa).

Substituting values into the equation, we obtain the following:

n = [2(1.96 = 0.84)^2^ (10.12)^2^] 9.26 ^2^ = [2 × (2.80)^2^ × 102.41] ÷ 85.71 ≈ 18.7.

This equals 19 specimens per group. To account for a potential 10% specimen loss, the sample size was increased to 20 per group. With 6 groups (3 irrigants × 2 activation methods), the total sample size is 120 specimens. Furthermore, if mean bond strength in the EDTA group=49.08, mean bond strength in the normal saline group=39.82, σ1=SD of bond strength in the EDTA group=10.19, and σ2=SD of bond strength in the normal saline group=10.04, for detecting a mean difference of 9.26, that is, Δ =49.08 − 39.82=9.26, we need K = 1, N = (17.33 × 17.33 + 2.81 × 2.81)(1.96 + 0.84)^2^, 23 × 23 = 18.71 = 19 patients in each group.

On the basis of previously published data [16], the mean push-out bond strength for the EDTA group should be 49.08 MPa (SD 10.19) and for the normal saline group it should be 39.82 MPa (SD 10.04). Using these values, a power analysis was performed assuming a 5% significance level (α=.05), 80% power (β=.20), and a mean difference of 9.26 MPa between groups.

### Group Allocation

The specimens were randomly allocated into 3 main groups based on the post space irrigant used, and further, each group was subdivided into 2 subgroups according to the activation method, as shown in Table 1.

**Table 1:** Sample size distribution.

Irrigant and activation method			Sample size, n
**Group A: normal saline**			
	Subgroup A1: ultrasonic activation	20	
	Subgroup A2: sonic activation	20	
**Group B: 5% calcium hypochlorite**			
	Subgroup B1: ultrasonic activation	20	
	Subgroup B2: sonic activation	20	
**Group C: 17% EDTA^a^**			
	Subgroup C1: ultrasonic activation	20	
	Subgroup C2: with sonic activation	20	

^a^EDTA: ethylenediaminetetraacetic acid.

### Recruitment and Procedure

For this in vitro investigation, a total of 120 healthy, recently excised human mandibular premolars of comparable size will be chosen. The teeth will be stored in 0.1% thymol solution at 4 °C until use to prevent microbial growth and preserve dentin integrity. Each specimen will be standardized to a 14-mm root length after the crowns are removed with a water-cooled diamond disk.

To verify working length and canal patency, a #10 K-file (Mani Inc) will be used. Using the ProTaper Universal rotary system (Dentsply) up to the F3 finishing file, canal preparation will adhere to the crown-down procedure. Following each filing, canals will be irrigated with 2 mL of distilled water and 5% sodium hypochlorite during instrumentation. Upon completion of instrumentation, a final flush will be given using 5 mL of 17% EDTA.

The single-cone obturation technique will be used with Diadent Dia-ProSeal sealer. The coronal 10 mm of the canal will be ready for fiber post implantation, and an apical 4 mm of gutta-percha (Dentsply Sirona) will be kept in the canal to ensure a sufficient apical seal. The coronal gutta-percha will be extracted using Peeso reamers (Mani Inc; 28 mm).

Following that, specimens will be randomly split into 3 major groups according to the type of post space irrigant, as described in Table 1. Sodium hypochlorite will be used during instrumentation for its established cleaning properties, whereas calcium hypochlorite will be tested as a post space irrigant to assess its effect on bond strength during post cementation.

Sonic activation will be done using the SuperEndo Activator, operating at frequencies between 1 and 6 kHz and fitted with flexible polymer tips appropriate for post space irrigation. Ultrasonic activation will be done using the Woodpecker UDS-K ultrasonic unit, operating at a frequency of approximately 25 to 30 kHz, with a size #20 noncutting tapered ultrasonic file.

The sonic activation frequency (1-6 kHz) was chosen according to the manufacturer’s specifications for the SuperEndo Activator device and previous studies showing the effectiveness of this range in agitating irrigants within post spaces. The ultrasonic activation frequency (approximately 25-30 kHz) reflects the operational parameters of the Woodpecker UDS-K ultrasonic unit, supported by literature reporting optimal cavitation and acoustic streaming effects at these frequencies.

Each post space will be irrigated with 5 mL of the allotted irrigant for 60 seconds. This activation time of 60 seconds was selected based on findings from Saber et al [16], Rödig et al [13], and Haupt et al [18], which demonstrated that this duration effectively removes the smear layer and enhances irrigant penetration without causing dentin erosion or excessive structural alteration. After irrigation, distilled water will be used to rinse the canals, and absorbent paper points will be used to dry them [16,18].

In compliance with the manufacturer’s instructions, fiber posts (Dentsply RelyX Fiber Posts, size #2 [diameter 1.5 mm and length 20 mm]) will be luted using a dual-cure resin cement (Dentsply Calibra Universal). Standardization of canal diameter and post space preparation to a uniform size (#2 post, 1.5-mm diameter) will be implemented to control for variability in post fit and bonded surface area, which could otherwise confound bond strength measurements. An etch-and-rinse technique will be used. The cement will be mixed and applied using a lentulo spiral. The post will be inserted with firm finger pressure for 40 seconds. Excess cement will be removed, followed by light curing for 40 seconds per side.

Three horizontal slices, each 1 mm thick, will be cut from each sample’s midroot region. The use of 1-mm–thick slices for push-out testing follows recommendations by Ferreira et al [12], as this thickness minimizes bending stresses during testing and provides more accurate measurement of interfacial bond strength. Each section will undergo mechanical testing after being checked for consistency. Universal testing equipment will be used to assess the strength of the push-out bond at a speed of 0.5 mm per minute, wherein a 1-mm–diameter metal pin will be positioned to supply force in an apical-to-coronal direction.

The maximum load at which the post dislodges must be recorded in newtons (N). The bond strength in MPa is calculated by dividing the recorded load (N) by the bonded interface area (A). The bonded interface area will be calculated using the following formula: A = π(R + r) √[h^2^ + (R – r)^2^], where R=coronal radius, r=apical radius, and h=slice thickness.

### Blinding

To minimize operator and measurement bias, specimen preparation, irrigation, and bond strength testing will be performed by different trained investigators who are blinded to the group allocations. The group codes will be concealed during mechanical testing and data entry.

### Interexaminer and Intraexaminer Reliability

To ensure measurement consistency and validity, 10% of the samples will be randomly selected and retested by the same examiner and by a second examiner. The intraexaminer and interexaminer reliability will be assessed using intraclass correlation coefficients, with values >0.80 considered acceptable.

### Ethical Considerations

Ethics approval for this study was obtained from the institutional ethics committee of Datta Meghe Institute of Higher Education and Research (IEC/2025/543).

All extracted teeth were obtained from the department of oral surgery as biological waste tissue following routine extractions performed for therapeutic reasons unrelated to this study. The specimens were anonymized before collection, with no patient identifiers recorded, and were stored in accordance with institutional biosafety protocols. As the specimens constituted waste tissue, individual informed consent was waived by the ethics committee; however, all procedures complied with national and institutional guidelines for the ethical handling and disposal of human-derived materials.

### Statistical Analysis

Statistical analysis will use both descriptive and inferential statistical methods, including the chi-square test, Student paired and unpaired *t* tests, 1-way ANOVA, and Tukey test. The analysis will be conducted using SPSS (version 27.0; IBM Corp) and GraphPad Prism (version 7.0; GraphPad Software Inc), with a significance level of *P*<.05. Descriptive statistics (including mean, SD, and 95% CI) will be calculated for each group. Differences in mean push-out bond strength (in MPa) among the 6 groups will be assessed using 1-way ANOVA. Post hoc comparisons will be performed using the Tukey honestly significant difference test to control for type I errors associated with multiple comparisons. A significance level of *P*<.05 will be considered statistically significant.

### Outcome Measure

#### Primary Outcome Measure

The primary outcome of this study is to compare the effects of sonic and ultrasonic activation of 3 post space irrigants—5% calcium hypochlorite, 17% EDTA, and saline—on the push-out bond strength of fiber posts luted with dual-cure resin cement in extracted human mandibular single-rooted premolars.

#### Secondary Outcome Measures

The secondary outcomes of this study are as follows:

- To assess how the push-out bond strength of fiber posts luted with dual-cure resin cement is affected by the sonic activation of normal saline, 5% calcium hypochlorite, and 17% EDTA.
- To assess how the push-out bond strength of fiber posts luted with dual-cure resin cement is affected by the ultrasonic activation of normal saline, 5% calcium hypochlorite, and 17% EDTA.
- To contrast the push-out bond strength attained for each irrigant using sonic and ultrasonic activation.
- To ascertain which combination of irrigant and activation offers the strongest push-out bond.

## Results

At the time of submission, ethics approval has been obtained (on February 7, 2025), and the study is self-funded. Sample collection commenced in June 2025, with 68 extracted mandibular premolars obtained to date. Specimen preparation is underway, and data collection is expected to be completed by March 2026. Data analysis is planned for November 2025, with results anticipated to be available for publication in early 2026.

## Discussion

### Anticipated Findings

In this in vitro study, the effects of sonic and ultrasonic activation of 3 post space irrigants—5% calcium hypochlorite, 17% EDTA, and saline—on the push-out bond strength of fiber posts luted with dual-cure resin cement will be investigated. The findings may indicate that both activation method and irrigant type affect adhesion to root canal dentin. It is hypothesized that the chelating action of EDTA may facilitate better bonding by efficiently eliminating the smear layer and revealing dentinal tubules [19]. If this is observed, such a finding would be consistent with the findings of Saber et al [16], who reported that EDTA significantly enhanced bonding strength compared with saline.

Additionally, 5% calcium hypochlorite is expected to strengthen the connection. This might be because calcium-containing crystals develop, which enhance chemical reactivity and promote bonding [17]. Saline, which has no chemical reactivity, is expected to produce the lowest bond strength [17].

An important factor is irrigant activation. On the basis of previous reports, ultrasonic activation is expected to produce a stronger bond than sonic activation due to enhanced cavitation and acoustic streaming. This is explained by ultrasonic cavitation and sonic streaming effects, which improve irrigant penetration and smear layer removal [16]. Despite being less potent, sonic activation is expected to outperform nonactivated irrigation and, because of its conservative nature, might be better in some therapeutic situations. This would be consistent with the findings of Marchese et al [19], who observed that EDTA was superior to other irrigants for post space cleaning.

To maximize post retention, this study will highlight the significance of using efficient irrigants and applying ultrasonic stimulation. In teeth that have received endodontic treatment, such procedures may lessen debonding and enhance clinical results.

The results might not accurately reflect intraoral circumstances because this study was conducted in vitro. Long-term aging, fatigue loading, and advanced imaging should all be included in future research to assess the interfacial properties and durability of the bonded post-dentin complex. While statistically significant differences in MPa values may be observed, their clinical significance should be interpreted with caution. Literature suggests that even modest increases in bond strength can enhance post retention and reduce debonding, potentially improving clinical longevity. However, long-term survival data will be required to validate these effects in vivo.

### Limitations

Although this study will provide controlled data on push-out bond strength, it is limited by the absence of aging simulations, such as thermocycling or mechanical fatigue loading. Future studies should incorporate cyclic loading and long-term water storage to better replicate intraoral conditions and evaluate the durability of the bonded interface over time.

A nonactivated control group for each irrigant was not included, as the focus was to compare activation techniques. Future studies may benefit from incorporating control groups to assess absolute efficacy compared with baseline irrigation. No failure mode analysis (cohesive, adhesive, or mixed) was performed; this has been proposed as a secondary analysis for future studies.

The study did not use scanning electron microscopy or confocal laser scanning microscopy to confirm smear layer removal, which limits direct correlation with bond strength outcomes. Such analyses are planned for inclusion in the secondary phase of the study. While calcium hypochlorite adds novelty, the exclusion of more recent irrigants, such as maleic acid or nanoparticle-based solutions, limits broader clinical relevance. These agents will be considered in subsequent research phases to enhance clinical relevance.

## Abbreviations

EDTA: ethylenediaminetetraacetic acid
MPa: megapascals
